# A novel application of hematite precipitation for high effective separation of Fe from Nd-Fe-B scrap

**DOI:** 10.1038/s41598-019-54896-3

**Published:** 2019-12-04

**Authors:** Xue Lin, Zhan Qu, Yu Chen, Ruinan Jin, Ting Su, Yang Yu, Suiyi Zhu, Mingxin Huo, Juwei Peng, Zhaofeng Wang

**Affiliations:** 1 School of Environment, Northeast Normal University, Changchun, 130117 China; 2 Jilin Institute of Forestry Survey and Design, Changchun, 130022 China; 3 Northeast Electric Power Design Institute Co. Ltd., Changchun, 130021 China; 4 Guangdong Shouhui Lantian Engineering and Technology Co. Ltd., Guangzhou, 510075 China; 5 Office of Qingyang Sponge City Construction and Management, Qingyang, Gansu 745099 China

**Keywords:** Environmental sciences, Engineering

## Abstract

Rare earths, e.g. neodymium (Nd), praseodymium (Pr) and dysprosium (Dy), are abundant in the rare earth sintered magnet scrap (Nd-Fe-B scrap), but their recycling is tedious and costly due to the high content of impurity Fe. Herein, a novel approach was developed to effectively recycle rare earths from the scrap via an integrated acid dissolution and hematite precipitation method. The scrap contained 63.4% Fe, 21.6% Nd, 8.1% Pr and 3.9% Dy. It was dissolved in nitric, hydrochloric and sulfuric acids, separately. Nearly all impurity Fe in the scrap was converted to Fe^3+^ in nitric acid but was converted to Fe^2+^ in hydrochloric and sulfuric acids. After hydrothermal treatment, the rare earths in the three acids were almost unchanged. From nitric acid, 77.6% of total Fe was removed, but total Fe was not from the hydrochloric and sulfuric acids. By adding glucose, the removal of total Fe was further increased to 99.7% in nitric acid, and 97% of rare earths remained. The major mechanism underlying total Fe removal in nitric acid was the hydrolysis of Fe^3+^ into hematite, which was promoted by the consumption of nitrate during glucose oxidation. This method effectively recycled rare metals from the waste Nd-Fe-B scrap and showed great potential for industrial application.

## Introduction

Rare earth sintered magnet scrap (Nd-Fe-B scrap) was generated in the production process of magnet, luminescent materials and catalysis^1,2^. The scrap contained approximately 30% of rare earths, including neodymium (Nd), praseodymium (Pr), dysprosium (Dy) and terbium (Tb)^3^, and 50%–65% of impurity Fe. The content of rare earths in the scrap was higher than in monazite (25 wt.%)^4^, amphibole (5 wt.%)^5^, and phosphorus (1.8–2.0 wt.%)^6^ ores. Thus, the scrap was an important resource for rare earth recovery.

Many approaches have been developed for rare earth recovery, which was generally initiated by dissolving the scrap in acids, such as sulfuric, hydrochloric and nitric acids. After dissolution, rare earths in acids were recycled in two ways. One was extraction by solvents, e.g. N,N-dioctyldiglycolamic acid^7,8^, together with complexes of D2EHPA^9^, PC88A^10^ and Cynaex572^11^. The extraction agents have high selectivity to rare earth and can effectively recycle rare earths from acids via tedious stratification. Following the dissolving of impurity Fe in scrap, the generated Fe^2+^/Fe^3+^, an active cation, reacts with the extraction reagent. This process leads to the accumulation of Fe^2+^/Fe^3+^ with repeated use of the extraction reagent, thereby reducing the efficiency of rare earth extraction and increases the cost^11^. The other way was precipitation of rare earths or Fe^2+^/Fe^3+^ by adjusting pH and/or adding a precipitant, such as oxalic acid^12^ and sodium sulfite^13^. Vander *et al*. reported that rare earths were precipitated by adding oxalic acid at the pH range of 2–2.5 after the dissolution of scrap in hydrochloric acid^12^, but Fe^2+^ reacted with oxalate acid to form precipitates of Fe^2+^ oxalate, which added impurity into the rare earth precipitates. Moreover, Fe^3+^ precipitated spontaneously with pH >2, and when the pH was increased to 4, about 99% of the dissolved Fe^3+^ from scrap was removed from the nitric acid solution^14^. During Fe^3+^ precipitation, it was hydrolysed rapidly to Fe^3+^ oxyhydroxide, in which one Fe atom coordinated with six hydrogen groups^15–17^. Therefore, the formed Fe^3+^ oxyhydroxide generated abundant hydrogen groups, in which rare earths could be coordinated, thereby resulting in low levels of dissolved rare earths in the solution.

When Fe^3+^ oxyhydroxide was converted to the well-crystallised Fe oxides, the two adjacent Fe-OH bonds on Fe^3+^ oxyhydroxide were dehydrated to form the Fe-O-Fe bond^18,19^, and the average number of coordination sites on Fe^3+^ oxyhydroxide decreased^20,21^, thereby subsequently reducing the precipitation of rare earths. He *et al*. reported that 90.7% of Fe^3+^ was eliminated as hematite when the Fe^3+^/Zn^2+^-bearing sulfuric acid solution was hydrothermally treated at 210 °C for 2 h with the addition of H_2_O_2_^22^. Despite the effective removal of Fe^3+^, the Fe^3+^ residual was still high (nearly 1,500 mg/L)^23^ and needed to be removed before rare earth extraction.

In this study, an integrated acid dissolution and hematite precipitation method was developed for the effective removal of the impurity Fe from scrap. After the scrap’s dissolution in nitric acid, 99.7% of total Fe was hydrothermally converted to hematite with the addition of glucose. Meanwhile, more than 97.1% of rare earths remained. This is the first report on the effective removal of impurity Fe from a rare earth-bearing solution with high rare earth retention.

## Results and Discussion

**Figure 1:**
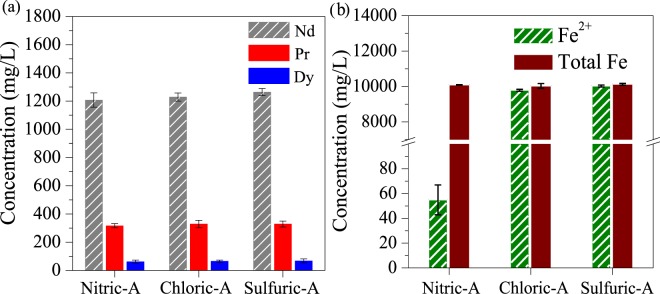
The concentrations of (**a**) rare earths, (**b**) total Fe and Fe^2+^ in the three acids.

After the scrap was dissolved in the nitric, hydrochloric and sulfuric acids, the generated acidic solutions were designated as Nitric-A, Chloric-A and Sulfuric-A, respectively. The concentrations of rare earths and total Fe (including Fe^2+^ and Fe^3+^) were similar in the three acids, as shown in Fig. 1(a,b). However, in Nitric-A, Fe^2+^ was only 54.9 mg/L, whereas Fe^3+^ was about 10,038 mg/L, as shown in Fig. 1(b), thereby indicating that Fe^3+^ predominated in the total Fe in Nitric-A. In comparison with Nitric-A, Fe^2+^ was approximately 10,000 mg/L in both Chloric-A and Sulfuric-A, as shown in Fig. 1(b), thereby suggesting that Fe^2+^ was rich in Chloric-A and Sulfuric-A due to the lack of oxidising agent (e.g. nitrate).

**Figure 2:**
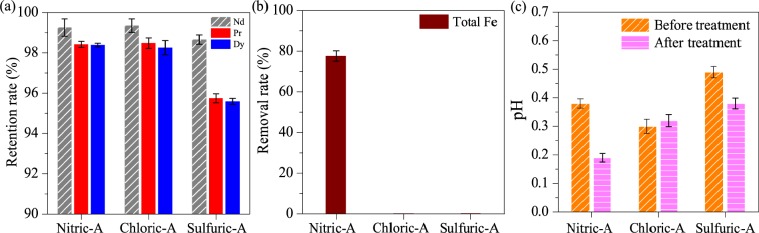
(**a**) Retention rate of Nd, Pr and Dy, (**b**) removal rate of total Fe after hydrothermal treatment, and (**c**) pH value of the three acids before and after hydrothermal treatment.

**Figure 3:**
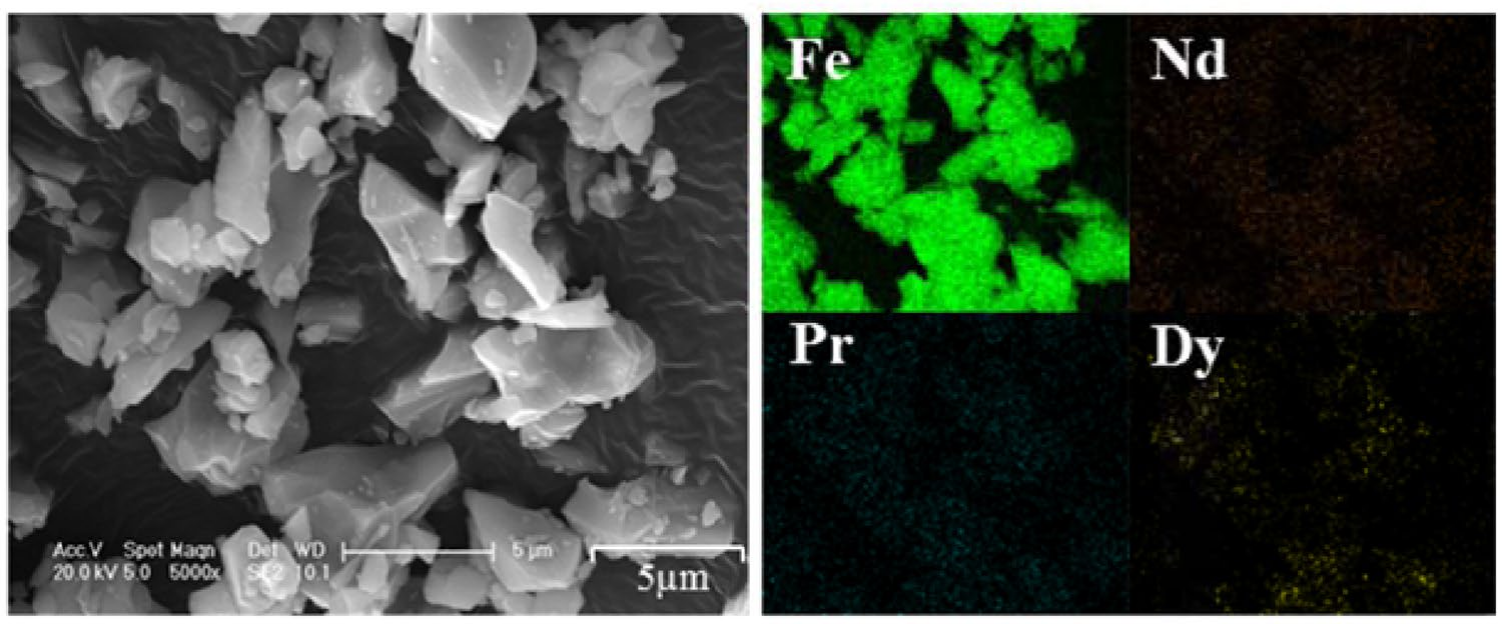
SEM image and EDS mapping of the Fe-bearing particles generated in nitric acid after scrap dissolution.

**Figure 4:**
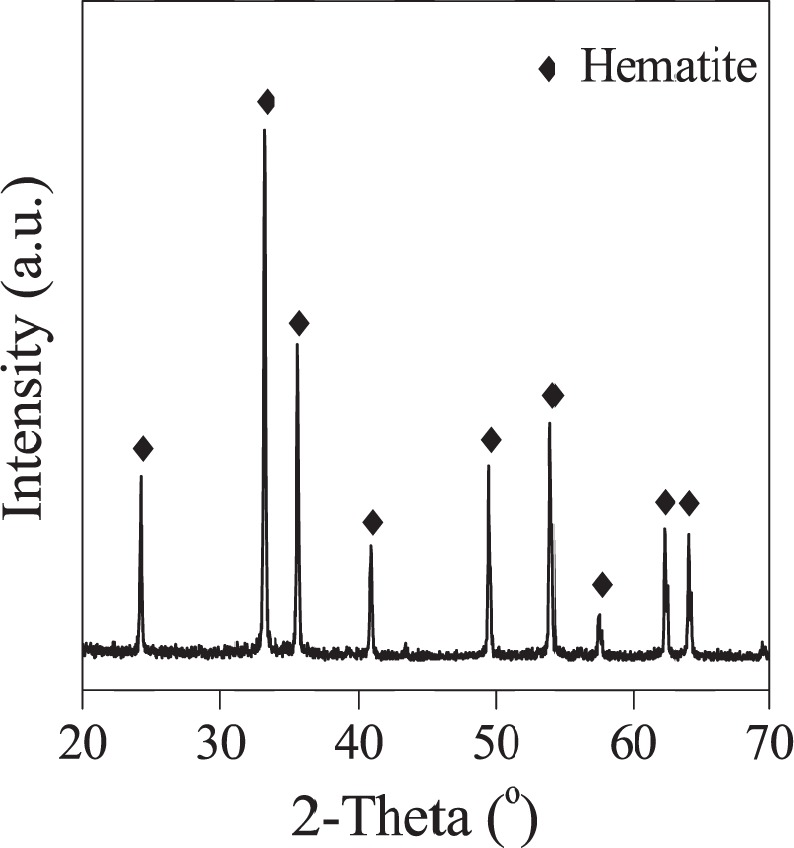
XRD pattern of the Fe-bearing particles generated in nitric acid after scrap dissolution.

After hydrothermal treatment, the concentrations of rare earths were almost unchanged in the three acids, as shown in Fig. 2(a). However, in Nitric-A, the total Fe concentration decreased from 10,093 mg/L to 2,257 mg/L, corresponding to 77.6% of the total Fe removal rate, as shown in Fig. 2(b). Meanwhile, the solution pH slightly decreased from 0.38 to 0.19, as shown in Fig. 2(c), due to the generation of H^+^ from the hydrolysis of Fe^3+^. The hydrolysed Fe^3+^ was in irregular form with the uniform distribution of element Fe and sparse distributions of Nd, Pr and Dy (Fig. 3), demonstrating that element Fe was dominant in the generated particles. Moreover, only indicative peaks of hematite (JSCPDS 33-0664) were observed in the curve of the generated particles (Fig. 4), indicating that Fe^3+^ was hydrolysed in the form of well crystallised hematite. Compared with Nitric-A, the total Fe concentrations in Chloric-A and Sulfuric-A were constant, as shown in Fig. 2(b), suggesting that the oxidation and hydrolysis of Fe^2+^ did not occur.

**Figure 5:**
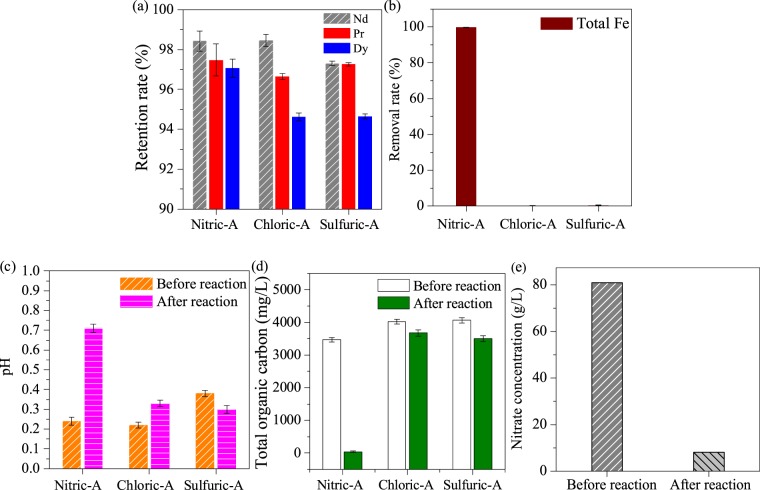
(**a**) Retention rates of Nd, Pr and Dy and (**b**) removal rate of total Fe after hydrothermal treatment with the addition of glucose; the variation of (**c**) pH and (**d**) total organic carbon before and after reaction; and (**e**) nitrate concentration in Nitric-A.

**Figure 6:**
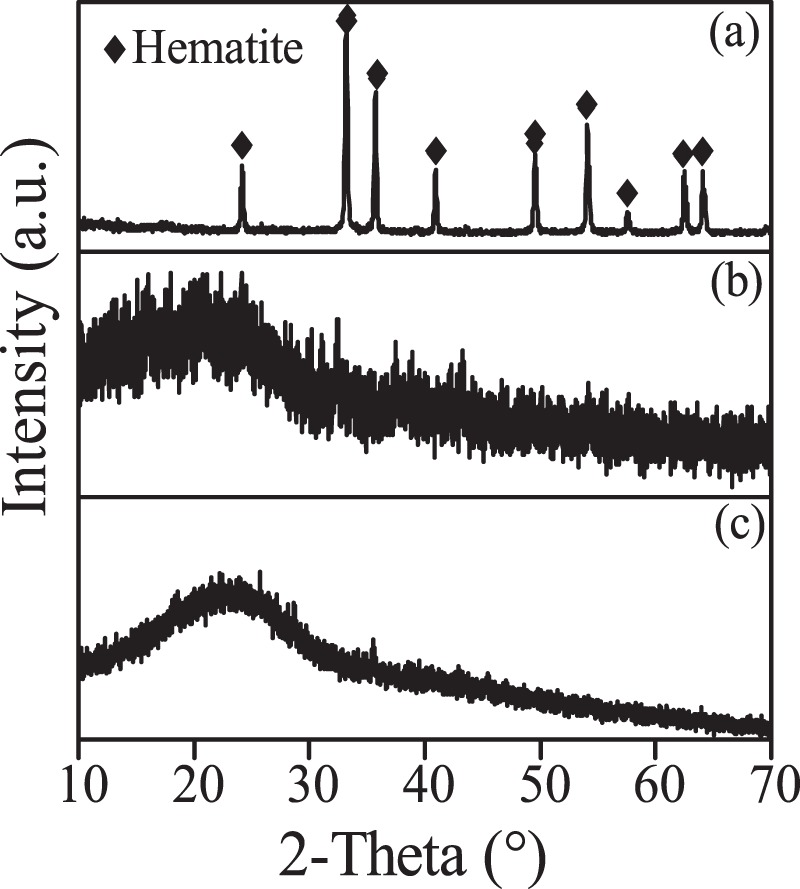
XRD patterns of the precipitates in the (**a**) nitric acid, (**b**) hydrochloric acid and (**c**) sulfuric acid after hydrothermal treatment with the addition of glucose.

**Figure 7:**
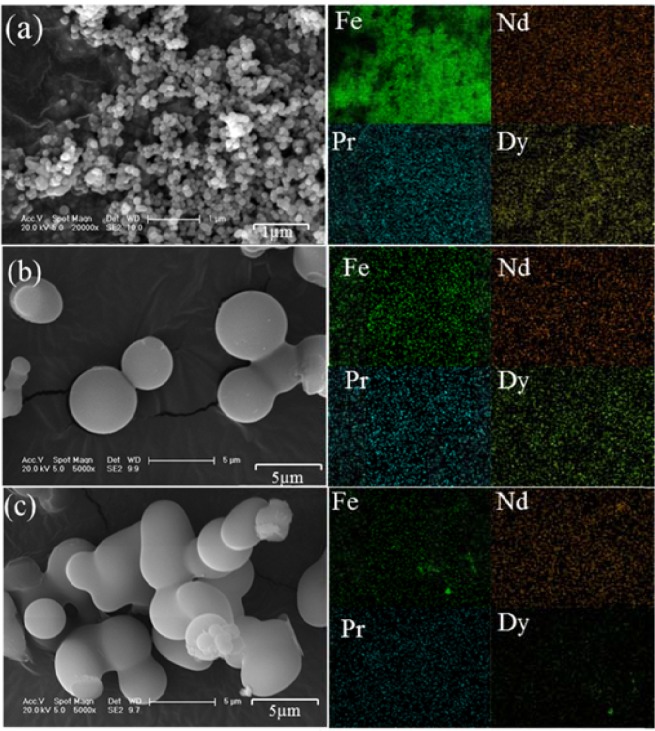
SEM images and EDS mappings of the precipitates generated in the (**a**) nitric acid, (**b**) hydrochloric acid and (**c**) sulfuric acid after hydrothermal treatment with the addition of glucose.

To further remove the total Fe from Nitric-A, glucose was introduced. Glucose’s efficiency to remove Fe is shown in Fig. 5. After hydrothermal treatment, the retention rates of rare earths were 98.4% for Nd, 97.5% for Pr and 97.1% for Dy, as shown in Fig. 5(a), and these rates were similar to those obtained without glucose (Fig. 2(a)). However, the removal rate of total Fe increased to 99.6% (Fig. 5(b)), much higher than that without glucose (Fig. 2(b)), indicating that glucose was important factor for total Fe removal without losing rare earths. With glucose, the total Fe was removed as hematite particles (Fig. 6(a)), similar to the product generated without glucose (Fig. 4), but with the average diameter of 80–100 nm, as shown in Fig. 7(a). During the process, the pH increased slightly from 0.24 to 0.71 (Fig. 5(c)), whereas total organic carbon dramatically decreased from 3,465 mg/L to 39.2 mg/L (Fig. 5(d)). Moreover, the nitrate concentration considerably decreased from 80.9 g/L to 8.08 g/L (Fig. 5(e)). These findings demonstrated that redox reaction occurred between glucose and nitrate, in which abundant glucose was oxidised by nitrate to CO_2_ and H_2_O with the consumption of H^+^.

In comparison with Nitric-A, no apparent change in total Fe concentration was observed in Chloric-A and Sulfuric-A after hydrothermal treatment with glucose, and only a few spherical particles with average diameters of 3–5 μm (Fig. 7(b,c)) were precipitated. The spherical particles showed an extremely broad XRD peak at 2θ = 23.2° (Fig. 6(b,c)) that probably belonged to the carbon sphere generated from the dehydration and polymerisation of glucose, similar to the hydrothermal product of glucose reported by Mi *et al*.^24^.

This approach exhibited a high removal rate (99.6%) of total Fe in the recycling of rare metals from the rare earth-bearing scrap. This rate was higher than that obtained through other reported processes, such as the complex leaching and electrolysis process with addition of H_2_SO_4_ and MnO_2_^25^, the extraction processwithtri-n-butyl phosphate(TBP)and tricaprylmethylammonium nitrate ([A336][NO_3_])^26^, the mechano-chemical treatment with HCl and (COOH)_2_^27^ and selective leaching with nitric acid^2^, sulfuric acid^28^ and ascorbic acid^29^ (Table 1).

**Table 1:** Comparison of the removal rate of total Fe and the retention rate of rare earths.

Method	Auxiliary reagent	Precipitation	Fe removal rate	Rare earth retention rate	Reference
Hematite precipitation method	Nitric acid and glucose	Hematite	99.6%	>97.1%	This study
Leaching and precipitation	Nitric acid and H_2_O_2_	Fe(OH)_3_ at pH = 2–3	98%	77%	^2^
Leaching and electrolysis process	Sulfuric acid and MnO_2_	Fe(OH)_3_ at pH >3	99%	77%	^25^
Extraction process	Nitric acid, TBP and [A336][NO_3_]	Fe(OH)_3_ at pH >4.5	99%	<92%	^26^
Mechano-chemical treatment	Hydrochloric acid and acetic acid	Nd_2_O_3_	<90%	95.3%	^27^
Selective leaching route	Sulfuric acid and NaOH	NdOOH and Nd(OH)_3_	Nearly 100%	75.41%	^28^
Selective leaching route	Ascorbic acid and phosphoric acid	NdPO_4_ at pH = 2.8	90%	99%	^29^

When the scrap was dissolved in hydrochloric and sulfuric acids separately, Fe^2+^-bearing solutions were generated. Fe^2+^ was stable in the two acids and not hydrolysed during the hydrothermal process, thereby resulting in low removal rates of total Fe. However, with nitric acid dissolution, Fe^3+^ was generated from the oxidation of impurity Fe in scrap by nitrate. The generated Fe^3+^ was further hydrothermally hydrolysed to hematite with generation of nitric acid when the temperature increased to 160 °C (Eq. (1))^30,31^. Hematite was well crystallised and had a protonated surface at pH <1^32^ in which the net surface charge on its surface was positive and blocked the adsorption of metal ions, such as Nd, Pr and Dy.

1 $$2F{e}^{3+}+6N{O}_{3}^{-}+3{H}_{2}O=F{e}_{2}{O}_{3}+6HN{O}_{3},$$

As the reaction continued, Fe^3+^ was hydrolysed to produce a large amount of nitric acid. An increase in nitric acid concentration decreased the solution pH from 0.38 to 0.19 (Fig. 2(c)) and shifted the hydrolysis equilibrium to the left (Eq. (1))^30^, resulting in a decrease of Fe^3+^ hydrolysis. Therefore, residual total Fe at a concentration of 2,257 mg/L was left in the solution (Fig. 2(b)).

Glucose was hydrothermally oxidised by nitrate to generate levulinic acid and 5-hydroxymethylfurfural, which were further oxidised to CO_2_ and H_2_O via Eq. (2). With the oxidisation of glucose, nitrate was hydrothermally reduced to N_2_, and its concentration apparently decreased from 80.9 g/L to 8.08 g/L (Fig. 5(e)), thereby promoting Fe^3+^ hydrolysis. Moreover, H^+^ was also involved in the redox reaction between glucose and nitrate (Eq. (2)). Thus, the solution pH increased from 0.19 to 0.71 (Fig. 5(c)), which further accelerated the formation of hematite^33^. During glucose oxidisation, the generated intermediates levulinic acid and 5-hydroxymethyl furfural were electrostatic adsorbed on the positively charged surface of hematite particles, thereby inhibiting the aggregation and crystal growth of hematite particles^34^ and resulting in smaller hematite particle sizes than without glucose.

2 $$\frac{5}{3}{C}_{6}{H}_{12}{O}_{6}+8N{O}_{3}^{-}+8{H}^{+}\to 10C{O}_{2}+4{N}_{2}+14{H}_{2}O,$$

## Materials and Methods

### Nd-Fe-B scrap

**Figure 8:**
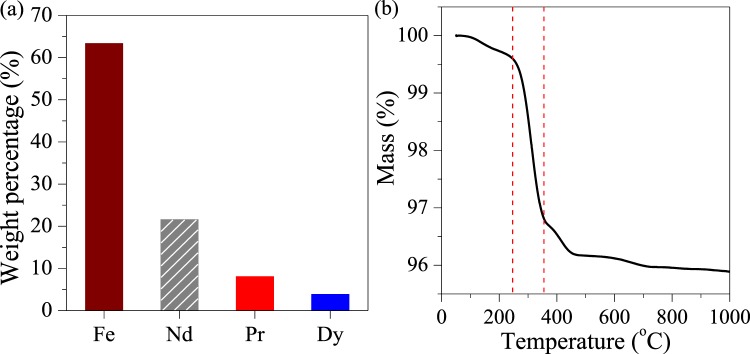
(**a**) Composition and (**b**) TGA plot of the Nd-Fe-B scrap.

Nd-Fe-B scrap was acquired from the calcinator of a local alumina refinery in Jilin, China. The scrap was ground to pass through a sieve with the mesh aperture of 1 mm and then dried overnight in a vacuum drying oven. The ground scrap was characterised by X-Ray fluorescence (XRF, XRF-1800, Shimadzu, Japan) and thermogravimetric analysis (TGA, 409PC, NetzschSTA, USA), and its major component was Fe (63.4%), Nd (21.6%), Pr (8.1%), Dy (3.9%) (Fig. 8(a)) and water (less than 4%, (Fig. 8(b)).

### Separation of Fe from Nd-Fe-B scrap

#### Dissolution of Nd-Fe-B scrap

The scrap was dissolved in acids as follows. Scrap (5 g) was dispersed in 250 mL of 3 M nitric acid under constantly stirring at 150 rpm overnight. A yellowish solution was generated and denoted as Nitric-A. The control experiments were also performed using 3 M hydrochloric and sulfuric acids, and the generated acidic solutions were designated as Chloric-A and Sulfuric-A, respectively.

#### Separation of Fe

Impurity Fe was separated from the acids via a one-step hydrothermal method. Nitric-A at 20 mL was transferred to a 50 mL Teflon kettle, hydrothermally treated at 160 °C for 10 h and cooled down to room temperature. The reddish particles generated at the bottom of kettle, were collected and vacuum-dried at 50 °C for 20 h before characterisation. The Chloric-A and Sulfuric-A were also hydrothermally treated, respectively, but no deposit was generated.

To further remove total Fe in Nitric-A, glucose was added at the glucose/total Fe molar ratio of 0.7. After agitating at 150 rpm for 5 min, a brownish suspension was generated. The suspension was hydrothermally treated according to the procedure described above. The obtained particles were collected for characterisation. Glucose was added to Chloric-A and Sulfuric-A with the same treatment as that used on Nitric-A, and the generated particles were collected and characterised separately.

Each experiment was performed thrice, and the averaged date was reported.

#### Characterisation

Total Fe and rare earths in the acids were analysed by inductively coupled plasma optical emission spectrometry (ICP-OES, Avio-200, Perkinelmer, USA). Fe^2+^ and Fe^3+^ in the acids were determined through the standard method^35^. Nitrate in the nitric acid was determined by ion chromatograph (881 pro, Metrohm, Switzerland). Total organic carbon and pH were measured by organic matter analyser (TOC 500, Shimadzu, Japan) and pH meter (S210-S, Mettler Toledo, USA). The crystallisation and morphology of the obtained particles were recorded by X-ray diffractometer (XRD, Rigaku, Rint2200, Japan) and scanning electron microscope (SEM, JSM-6400, JEOL, Japan), respectively.

## Conclusions

The scrap was dissolved in nitric, hydrochloric and sulfuric acids. Among these acids, nitric acid was optimal for dissolving the scrap, and nearly all the impurity Fe in the scrap was converted into Fe^3+^. In the following hydrothermal treatment, 77.6% of the total Fe in nitric acid was removed as hematite particles. By adding glucose, the total Fe removal rate was further increased from 77.6% to 99.6%, whereas over 97.1% of the rare earths in nitric acid remained. In nitric acid dissolution, the generated Fe^3+^ was hydrothermally hydrolysed to form hematite, which was promoted by the reduction of nitrate with glucose. With this method, total Fe was efficiently separated from nitric acid while retaining a high concentration of rare earths. This method has great potential for use in recovering rare earths from Nd-Fe-B scrap.

## References

[1] Habashi F (2003). Extractive metallurgy of rare earths. Canadian metallurgical quarterly..

[2] Rabatho JP (2013). Recovery of Nd and Dy from rare earth magnetic waste sludge by hydrometall- urgical process. Journal of Material Cycles and Waste Management..

[3] Machida K (2001). Resources and recycle for rare earth magnets. Rare Earth..

[4] Frietsch R, Perdahl JA (1995). Rare earth elements in apatite and magnetite in Kiruna-type iron ores and some other iron ore types. Ore Geology Reviews..

[5] Aide M, Aide. C (2012). Rare earth elements: their importance in understanding soil genesis. International Scholarly Research Notices..

[6] Clark, A. Mineralogy of the rare earth elements. In “Rare earth element geochemistry”, Hender-son, P., ed. *Elsevier Science Publishers BV*, *Amsterdam*, *Oxford*, *New York*, *Tokyo*. **33**, 61 (1984).

[7] Shimojo K (2007). Extraction behavior and separation of lanthanides with a diglycol amic acid deriv- ative and a nitrogen-donor ligand. Analytical Sciences..

[8] Gupta CK, Krishnamurthy N (2013). Extractive metallurgy of rare earths. Metallurgical Reviews..

[9] Gergoric M (2017). Separation of heavy rare-earth elements from light rare-earth elements via solvent extraction from a neodymium magnet leachate and the effects of diluents. Journal of Sustainable M-etallurgy..

[10] Nakamura T, Nishihama S, Yoshizuka K (2007). Separation and recovery process for rare earth metals from fluorescence material wastes using solvent extraction. Solvent Extraction Research and Devel- opment..

[11] Pavón S (2018). Neodymium recovery from NdFeB magnet wastes using Primene 81R· Cyanex 572 IL by solvent extraction. Journal of environmental management..

[12] Vander Hoogerstraete T (2014). From NdFeB magnets towards the rare-earth oxides: a recycling pr- ocess consuming only oxalic acid. RSC Advances..

[13] Onoda H, Fukatsu R (2016). Synthesis of neodymium phosphate from iron-neodymium solution using s- odium sulfite. Journal of Environmental Chemical Engineering..

[14] Mehmet (2017). Hydrometallurgical recycling of NdFeB magnets: Complete leaching, iron removal and electrolysis. Journal of rare earths..

[15] Jianmin Z (1994). Ferrihydrite: surface structure and its effects on phase transformation. Clays and Clay Minerals..

[16] Zhu S (2018). Hydrothermal synthesis of a magnetic adsorbent from wasted iron mud for effective r- emoval of heavy metals from smelting wastewater. Environmental Science and Pollution Research..

[17] Ma J (2018). Distinct effect of humic acid on ferrihydrite colloid-facilitated transport of arsenic in saturated media at different pH. Chemosphere..

[18] Ma J (2018). Enhanced transport of ferrihydrite colloid by chainshaped humic acid colloid in satura- ted porous media. Science of the Total Environment..

[19] Zhu S (2015). A novel conversion of the groundwater treatment sludge to magnetic particles for the adsorption of methylene blue. Journal of Hazardous Materials..

[20] Ma J (2015). Arsenic adsorption and its fractions on aquifer sediment: effect of pH, arsenic species, and iron/manganese minerals. Water, Air, & Soil Pollution..

[21] Zhu S (2019). Valorization of manganese-containing groundwater treatment sludge by preparing ma- gnetic adsorbent for Cu(II) adsorption. Journal of Environmental Management..

[22] He J (2010). Separation of zinc and iron in sulfate system with hydrothermal method and preparation of ferric oxide powder for soft magnetic ferrite. Mining and Metallurgical Engineering..

[23] Kelen, T. Method of preventing the deposition of radioactive corrosion products in nuclear plants. *Google Patents* (1999).

[24] Mi Y (2008). Synthesis of carbon micro-spheres by a glucose hydrothermal method. Materials Lette- rs..

[25] Önal MAR (2017). Hydrometallurgical recycling of NdFeB magnets: Complete leaching, iron rem- oval and electrolysis. Journal of Rare Earths..

[26] Kikuchi Y, Matsumiya. M, Kawakami. S (2014). Extraction of rare earth ions from Nd-Fe-B magnet wa- stes with TBP in tricaprylmethylammonium nitrate. Solvent Extraction Research and Development, Japan..

[27] Sasai R, Shimamura N (2016). Technique for recovering rare-earth metals from spent sintered Nd-Fe-B magnets without external heating. Journal of Asian Ceramic Societies..

[28] Lee CH (2013). Selective leaching process for neodymium recovery from scrap Nd-Fe-B magnet. Metallurgical and Materials Transactions A..

[29] Onoda H, Nakamura R (2014). Recovery of neodymium from an iron–neodymium solution using phosp- horic acid. Journal of Environmental Chemical Engineering..

[30] Shang Y, Van Weert. G (1993). Iron control in nitrate hydrometallurgy by autoclave hydrolysis of iron (III) nitrate. Hydrometallurgy..

[31] Wieczorek-Ciurowa K, Kozak. A (1999). The thermal decomposition of Fe(NO_3_)_3_ · 9H_2_O. Journal of Th- emal Analysis and Calorimetry..

[32] Wang P, Lo IM (2009). Synthesis of mesoporous magnetic γ-Fe_2_O_3_ and its application to Cr(VI) remo- al from contaminated water. Water Research..

[33] Charlatchka R, Cambier P (2000). Influence of reducing conditions on solubility of trace metals in cont- aminated soils. Water, Air, and Soil Pollution..

[34] Esmaeili E (2011). Modified single-phase hematite nanoparticles via a facile approach for large scale synthesis. Chemical engineering journal..

[35] Wu J, Zhang H, Qiu J (2012). Degradation of Acid Orange 7 in aqueous solution by a novel electro/ Fe^2+^/peroxydisulfate process. Journal of Hazardous Materials..

